# Compound Fracture of Femoral Bone; Resection of Fractured Ends, Recovery

**Published:** 1892-09

**Authors:** R. Menger

**Affiliations:** San Antonio


For Daniel's Texas Medical Journal.

### COMPOUND FRACTURE OF FEMORAL BONE; RESECTION OF FRACTURE ENDS.—RECOVERY.

R. MENGER, M. D., SAN ANTONIO.

THE FOLLOWING case of femoral fracture is put on record on account of its early history and other interesting features.

In June this year, eight weeks ago, a German boy, aged nine years and eight months, was brought to San Antonio a distance of twenty-five miles, with his hip-bone sticking two inches through his pants. The boy had been with his parents at a picnic, and in endeavoring to get into the ambulance from behind the vehicle, the horses suddenly commenced to walk away, and the boy slipped with his left leg between the spokes of the wheel and, whilst still hanging with his hands to the ambulance, his leg was so long stretched and turned around by the wheel until the thigh bone broke through the flesh. When the boy was brought to town his clothing, saturated with blood, could only be removed, under chloroform, by cutting it open with a sharp knife. The upper fracture end of femur was protruding fully three inches through the muscles, which were torn apart and protruded through the large skin opening; the entire laceration measuring five inches wide and four inches long, situated at the inner side and reaching from the summit to the base of the thigh. There also was extensive denudation of skin along the outside and inner border of the thigh and under the knee joint. From the appearance of the projecting and somewhat splintered fracture ends, it was deemed necsssary to resect these ends; and Drs. D. Berry and A. Graves, Jr., kindly assisted at the operation. Several subcutaneous whiskey and ether injections had to be made before and during the operation, on account of the very prostrated condition of the boy. After resection it was somewhat difcult to decide which plan was best to adopt for further treatment, on account of the skin abrasions, the skin being at some places torn off. As the boy's condition was very feeble, respiration down to six per minute, the leg was simply protected by two sand bags, laid on both sides of the thigh for a few days, and then I put the leg in a well padded gypsum bandage with a long outside splint, at the same time protecting the denuded skin surfaces with a strong bismuth salve, and leaving a large opening in the plaster bandage for the fracture wound. The boy now progressed fairly well up to about the second week, when he complained considerably of his leg, necessitating the removal of the plaster bandage (under chloroform), and I feared either gangrene or severe inflammation at the site of the skin abrations. Luckily, though, this was an error, as these places were granulating nicely and most of them were already healed. After chloroforming again, the leg was this time put in an extra prepared fracture box, a box with extra doors to get at the lacerated parts and so adjusted to a large plank that it reached from above the pelvis down to and above the toes. At the pelvic region the board had a large round opening (for the discharges), and was covered its entire length with oil cloth and the box partly filled with bran. For about four weeks this did very well; the large fracture wound granulated well after draining and dressing with aristol ointment twice a day; but it now was noticed that at several places, unavoidedly, the bran was soaked with the discharges from the granulating skin abrasions and the fracture wound; the fracture box was dispensed with; and as by this time the boy's condition in general was a great deal more favorable, the boy was again chloroformed, the fracture box removed and the leg kept quiet by placing two large and heavy sandbags along the leg, the outer one reaching from the shoulder down to the foot. Ossification now progressed favorably, although a great deal of trouble was yet had with several bed sores, etc.

The entire fracture wound is now healed up except a small opening which slightly discharges pus. The boy's general condition is very good, with only 1½ inch shortening of the leg,—certainly a good result, considering that besides the resected ends, there was loss of bone substance, done by the running wheels, which, with the resected bones, left about 2½ inches bone missing in continuity.

